# MMPI-2 Profile of French Transsexuals: The Role of Sociodemographic and Clinical Factors. A cross-sectional design

**DOI:** 10.1038/srep24281

**Published:** 2016-04-12

**Authors:** Mireille Bonierbale, Karine Baumstarck, Aurélie Maquigneau, Audrey Gorin-Lazard, Laurent Boyer, Anderson Loundou, Pascal Auquier, Christophe Lançon

**Affiliations:** 1 Department of Psychiatry, Sainte-Marguerite University Hospital, Assistance Publique-Hôpitaux de Marseille, 13009 Marseille, France; 2 EA 3279 Research Unit, Aix Marseille University & Assistance Publique-Hôpitaux de Marseille, 13005 Marseille, France

## Abstract

The assessment of co-existing psychological and psychiatric disorders is advocated in the Standards of Care for the health of transsexual people. This study aimed to determine the psychopathological characteristics of transsexuals based on a large sample of French individuals and to identify whether these characteristics differed according to the individual’s sociodemographic or clinical characteristics. The aim of this study was to determine the psychopathological characteristics of transsexuals from a large sample of French individuals and whether these differed by sociodemographic or clinical characteristics. This cross-sectional study was conducted in a French public university hospital. The inclusion criteria were 18 years or older, diagnosis of gender dysphoria, and eligibility for a standardized sex reassignment procedure. Personality characteristics were assessed using the Minnesota Multiphasic Personality Inventory 2 (MMPI-2). A total of 108 individuals provided a valid MMPI-2 between January 2007 and December 2010. The final sample had a median age of 31 years and included 54 (50%) Female-to-Male individuals. In multivariate models, hormonal therapy status was significantly related to the scales of MMPI-2 (Psychasthenia and Masculinity/Femininity). Personality assessment can help a multidisciplinary gender dysphoria team detect potential psychopathological factors of vulnerability.

An important area of research in gender dysphoria^1^, also known as transsexualism, involves the frequency and nature of psychopathology and the comorbidity of mental disorders. Information on psychological impairment may help health care providers identify the most appropriate clinical management for transsexuals individuals. The continued demand for appropriate treatment decisions for gender reassignment candidates underscores the need for further research to aid in establishing the nature and degree of psychopathology that these individuals are likely to experience.

As recommended in the 7^th^ version of the Standards of Care for the Health of Transsexual People^2^, mental health professionals should screen for these psychological characteristics and other mental health concerns and incorporate the identified concerns into the overall treatment plan. Existing studies have revealed contradictory results, highlighting either normative functioning^3^,^4^,^5^,^6^,^7^,^8^,^9^,^10^,^11^ or a high proportion of psychiatric comorbidity^12^,^13^,^14^,^15^,^16^. These discrepancies may the result of the following methodological differences. First, the studies included heterogeneous populations and often examined mixed clinical groups that included both transsexuals and other gender-nonconforming individuals, as well as individuals in different phases of the gender transition process. Second, psychological characteristics were established using varying psychometric measures, including different tests and structured psychiatric interviews. Although psychological distress may result from multiple stressors associated with sex reassignment, including frequent familial and social rejection, employment problems, and economic and legal difficulties^10^,^17^, few data are available. We provide information from a large homogeneous sample of transsexuals including those undergoing a standardized sex reassignment procedure, using a standardized and widely available personality test, the Minnesota Multiphasic Personality Inventory 2, for which norms are available.

This study aimed to determine the psychopathological characteristics of transsexuals based on a large sample of French individuals and to identify whether these characteristics differed according to the individuals sociodemographic or clinical characteristics.

## Methods

### Design

This study used a cross-sectional design and was conducted in the psychiatric department of a French public university teaching hospital (Marseille, France). In this department, individuals with gender dysphoria are managed in a special unit by a multidisciplinary team. This specific management is based on the chart of the French Society for Transsexual Management (Société Française d’Etudes et de Prise en Charge du Transsexualisme, SoFECT), which was recently created to improve coordination among the various professionals involved in caring for transsexual individuals (http://www.transsexualisme.info). Our gender team has adopted the guidelines of the International Standards of Care of the World Professional Association for Transgender Health^2^, which represents an international consensus on the psychiatric, psychological, medical, and surgical management of gender dysphoria. In France, six care units operate according to these guidelines. The study was approved by the local ethics committee (Comité de Protection des Personnes, Marseille, France, Number 07 070/2007-A01173-50). The procedures were carried out in accordance with the approved guideline, and all participants provided written informed consent.

### Sample selection

The inclusion criteria were as follows: age 18 years or older; a diagnosis of gender dysphoria according to the criteria of the Diagnostic and Statistical Manual (DSM-IV-TR)^18^; and eligibility for a standardized sex reassignment procedure^19^ following the agreement of a multidisciplinary team^20^. In France, the SoFECT chart has been developed according to the principals of WPATH and the French Health Authority^20^ and includes the following: a 12-month minimum multidisciplinary evaluation period to obtain a ruling on eligibility for sex reassignment. If conditions are satisfactory, hormonal therapy is initiated for a 12-month minimum period before sex reassignment surgery. The hormonal therapy proceeds with a reversible phase followed by an irreversible phase.

The initial sample consisted of 143 adults consecutively referred to the gender identity unit between 2007 and 2010. Evaluations were performed by a psychiatrist and a psychologist, both of whom were experienced in gender dysphoria management. Confirmation of eligibility for the sex reassignment procedure required the agreement of the two experts.

## Data collection

### The following data were collected

1. Sociodemographic information: medical files and interviews were used to report gender identity (male-to-female MtF/female-to-male FtM), age (younger: <median age; older: ≥median age), hormonal therapy (the patients were categorized into the hormonal therapy group if they had received physician-prescribed cross-sex hormones for a minimum of 3 months’ as a part of the sex reassignment procedure, corresponding to a minimal delay for expected physical changes^2^), onset age (early vs. late onset age, i.e., beginning before vs. after puberty^21^), and sexual orientation (same biological sex exclusively vs. others).

2. Personality test scores: personality was assessed using the Minnesota Multiphasic Personality Inventory 2 (MMPI-2), administered by a psychologist as part of a more extensive psychological evaluation. The MMPI-2 is a questionnaire widely used to assess psychopathological functioning^22^,^23^. The MMPI-2, a restandardization of the MMPI, is a 567-item true–false questionnaire. The inventory includes i) 3 validity scales: Lie (L), Infrequency (F), and Correction (K); ii) 10 clinical scales: Hypochondriasis (Hs), Depression (D), Hysteria (Hy), Psychopathic Deviate (Pd), Masculinity/Femininity (Mf), Paranoia (Pa), Psychasthenia (Pt), Schizophrenia (Sc), Hypomania (Ma), and Social Introversion (Si). French norms were available^24^. Raw scores were converted to uniform T scores relative to the normative data for the subject’s biological sex. Higher scores indicate less favourable functioning. The exclusion criteria for the MMPI-2 data analyses are in accordance with the French scoring manual: 1) Lie scale T-score above 80; 2) Frequency scale T-score above 110; and 3) Correction scale T-score above 80. For the clinical scales, a T-score of 50 can be interpreted as a score in the normal range, and a T-score of 65 or higher can be considered clinically significant.

### Statistical analysis

The MMPI-2 data were analysed using both means and standard deviations (T-scores) and the proportions of individuals with a score higher than 65 according to the MMPI-2 manual. Comparisons between sex reassignment surgery eligible and non-eligible patients were performed for biological sex, age, hormonal therapy status, and MMPI-2 scales. For each biological sex, the MMPI-2 scores were compared according to four variables of interest: age group (younger vs. older), hormonal therapy status (yes vs. no), onset age (early vs. late onset age), and sexual orientation (same biological sex vs. others), using the two-tailed Mann-Whitney t-test for independent samples. The proportions of individuals with scores ≥65 were compared among the subgroups using chi-squared or Fisher’s exact tests. For each individual, the number of scales (excluding the Masculinity/Femininity scale which reflects gender dysphoria) on which he or she scored in the clinical range was calculated. To determine potential variables of interest linked to the MMPI-2 scores (biological sex, age group, hormonal therapy status, onset age, and sexual orientation), logistic regression models were performed using a forward stepwise approach. The final models expressed the odds ratios (OR) and 95% confidence intervals (CI). All of the tests were two-sided. Statistical significance was defined as p < 0.05. Statistical analysis was performed using the SPSS version 15.0 software package (SPSS, Inc., Chicago, IL, USA).

## Results

### Sample

A total of 143 consecutive referrals for sex reassignment surgery were assessed between January 2007 and December 2010. Twenty-nine subjects were considered non-eligible for sex reassignment surgery by the multidisciplinary team and failed to meet the DSM-IV-R criteria. The 29 non-eligible subjects were significantly more often MtF than the 114 eligible subjects (79.3% vs. 50.9%, p < 0.01), and they less often reported receiving hormonal therapy. The proportion of scores over 65 for Hypochondriasis was significantly higher in the non-eligible group compared with the eligible group. According to the definitions in the French MMPI-2 manual, 2 (6.9%) non-eligible individuals and 6 (5.3%) eligible individuals presented an invalid test. These details are presented in Table 1.

**Figure 1:**
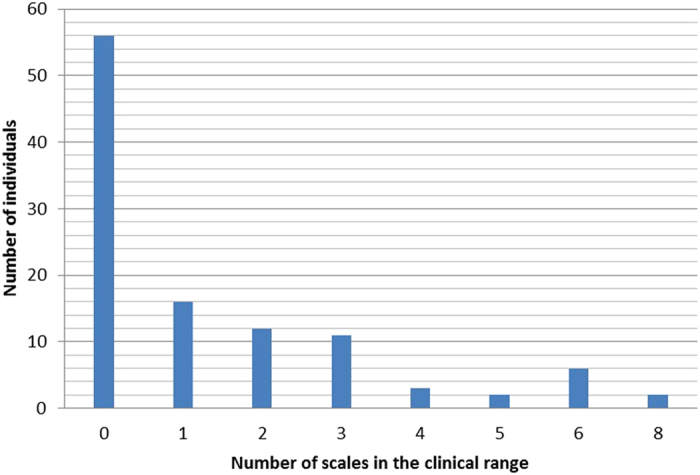
Proportions of individuals with of abnormal scores on MMPI scales*. *Excluding the Masculinity/Femininity scale.

Of the 114 eligible individuals, 108 had a valid MMPI. The final sample of 108 participants included 54 (50%) FtM individuals with a median age of 31 years (range 18–58). Fifty-two (49%) individuals received hormonal therapy (2 were missing data), 83 (78%) presented an early onset age (2 were missing data), and 83 (81%) reported a same-biological-sex sexual orientation (6 individuals did not provide this information). Approximately 48% (n = 52) of the individuals had at least one scale score (excluding the Masculinity/Femininity scale) in the clinical range (Fig. 1). The proportion of individuals in the clinical range was the lowest for the Social Introversion scale and the highest for the Masculinity/Femininity scale. All of the results are detailed in Tables 1 and 2.

The T-score means differed significantly between FtM and MtF individuals on 4 of the clinical scales (Hypochondriasis, Depression, Hysteria, and Paranoia), with higher scores for the MtF individuals (Table 2). The proportions of individuals in the clinical range were significantly higher in the MtF group compared with the FtM group for the Hypochondriasis and Depression scales.

### FtM individuals

None of the MMPI scores differed between the younger and the older groups. Because no FtMs reported a late onset age, and only one declared not having an exclusive sexual orientation with the same biological sex, no statistical tests were performed according to these 2 parameters. Individuals who were not receiving hormonal therapy had significantly higher scores on the Masculinity/Femininity and Schizophrenia scales. These results are provided in Table 3.

### MtF individuals

The older MtFs had significantly higher scores for Hypochondriasis scale compared with the younger MtFs. The individuals without hormonal therapy had significantly higher scores for Hypomania and a higher proportion of scores ≥65 on the Psychasthenia scale. The T-score means and proportions of scores ≥65 for all of the scales were not statistically higher for the individuals without hormonal therapy compared to the individuals with hormonal therapy. Compared with the late onset age transsexuals, the early onset age transsexuals had significantly higher T-score means for the Hypomania scale and higher proportions of scores ≥65 on the Social Introversion scale. When sexual orientation was exclusively geared toward the same biological sex, the individuals had significantly higher scores on the Hypomania scale and higher proportions of scores in the clinical range for the Schizophrenia scale. These results are provided in Table 4.

### Multivariate approach

The multivariate models showed no links between the MMPI-2 scores and age group, age at onset of transsexualism, and sexual orientation. Mtf individuals and individuals who were not receiving hormonal therapy were significantly more likely to be in the clinical range on the Psychasthenia scale (OR = 4.2, p = 0.05 and OR = 7.0, p < 0.01, respectively). Individuals without hormonal therapy also had a higher risk of being in the clinical range on the Masculinity/Femininity scale (OR = 2.8, p < 0.05). No other relationships were found for the other clinical scales (data not shown).

## Discussion

Although three previous studies^5^,^10^,^25^ have examined differences in MMPI-2 scores according to gender, age, and sexual orientation, to our knowledge, the present study was the first to report MMPI-2 scores for a large sample of French transsexuals according to their biological sex, age, hormonal therapy status, onset age, and sexual orientation.

The main finding of this study was that transsexual individuals present profiles that are relatively free of psychopathology. Several previous studies have described the psychological testing of transsexuals. In the oldest studies, the results were inconsistent. Although some studies have concluded that the MMPI profiles of the participants indicated serious psychological disturbances, depression, and interpersonal problems^12^,^26^,^27^,^28^,^29^, others have concluded the opposite^3^,^5^,^7^,^8^,^30^,^31^,^32^,^33^,^34^,^35^. The heterogeneity of the studied populations and the relatively small sample sizes of most of the studies could explain in part the contradictory findings. In the most recent studies, which were conducted with larger homogeneous samples of highly selected transsexuals (i.e., those who were eligible for a standardized sex reassignment procedure) and/or using the MMPI-2^4^,^7^,^10^,^25^, the results were consistent with ours and support the view that transsexualism is usually an isolated diagnosis^5^,^7^,^8^,^10^,^25^. The main message of these last studies was that transsexuals showed no evidence of psychopathology according to the MMPI-2 profile, and transsexualism can exist independent of other psychopathology. These results are consistent with our clinical experience and underline the fact that, contrary to the formerly widespread opinion, transsexuals do not have significant psychological disorders, and their demands for sex reassignment surgery are not symptoms of psychopathology.

Consistent with previous studies, the scales on which the highest proportions of individuals scored in the clinical range were Masculinity/Femininity and Psychopathic Deviate^3^,^5^,^28^,^31^,^36^,^37^. As expected^3^,^7^,^10^,^37^, the Masculinity/Femininity scale is where both biological men and women most frequently showed profiles in the clinical range^5^. This result clearly reflects the gender role discomfort these individuals experience in terms of their biological sex. Therefore, we can hypothesize that an individual may provide a normal or abnormal score depending on where he/she is in the course of the disorder. However, it is important to note that this psychological testing was not used to diagnose transsexualism or comorbidities of transgender but to provide a psychological profile that would be useful to the psychiatrist. Some authors have suggested that the MMPI scores of transsexual individuals should be interpreted using the normative data for the subject’s gender identity^5^,^28^,^36^. The Masculinity/Femininity scale scored for the biological sex is frequently abnormal but becomes normal when it is scored for the desired sex. The Masculinity/Femininity scale may be considered a reflection of the gender role dysphoria of these individuals. It is also often viewed as a nonclinical dimension of the MMPI^7^.

MtFs scored in the clinical range more often than FtMs on the Hypochondriasis and Depression scales. The literature shows contradictory results. Whereas similar studies did not find significant differences in the MMPI-2 profile according to sex^4^,^5^,^10^, other authors have tended to indicate that MtF individuals are more psychologically distressed than FtMs^3^,^25^,^26^,^37^,^38^. Some of these studies focused on different populations, such as participants with a late onset of the disorder, i.e., those who adapted to their biological sex during early life^26^, or gender dysphoric adolescents^38^. MtF individuals appeared more affected than FtMs on three other scales: Hypochondriasis, Depression and Hysteria. These scales can be considered neurotic-like, raising the question of whether MtFs are more neurotic than FtMs, because of stigma^39^. These results may indicate that, with regard to the body image concerns of MtF and FtM individuals, MtFs more often request visual changes to the body. The FtMs, in comparison with the MtFs, seem to pass more easily for the opposite gender^25^.

While the psychological functioning did not differ according to age for the FtMs, our results were as expected for the MtFs in terms of hypochondriasis, which is more frequent with older age^40^,^41^, mainly because of greater concerns about health problems. Because all the FtMs reported an early onset and almost all of them reported a same biological sex orientation, no comparisons could be made on this basis. Contrary to other studies^25^, we found worse psychological functioning in the MtFs with a same-biological sexual orientation in comparison with other cases. These findings should always be interpreted with caution because sexual orientation consists of various components and can change during a person’s lifetime^42^.

Not surprisingly, the no-hormonal therapy group functioned psychologically worse than the hormonal therapy group (the Schizophrenia and Masculinity/Femininity scales in the FtMs, and the Psychasthenia and Hypomania scales in the FtMs). We can hypothesize that transsexual individuals without hormone therapy tend to be centred on their own difficulties, depressed, and uncomfortable with other people. In contrast, hormonal therapy is often accompanied by a change in gender role, better self-confidence, greater comfort with others, and better social skills^43^,^44^. Furthermore, some authors have recently reported that initiating the hormonal treatment in untreated patients seemed to have a positive effect for reducing stress levels^45^ and more generally psychopathology^46^. By definition, individuals did and did not undergo hormonal therapy were not in the same phase of the sex reassignment procedure. The procedure may reinforce gender affirmation with better social recognition. In the same way, hormonal therapy, by inducing external physical modifications, can reduce the feelings of foreignness or strangeness that individuals sometimes report, thus explaining the scores found on the Schizophrenia scale which decrease. We can hypothesize that the absence of these different psychological disorders as a result of hormonal therapy may accelerate an individual’s potential eligibility in the sex reassignment procedure.

After accounting for biological sex, age group, onset age, and sexual orientation, the hormonal therapy status should affect the Masculinity/Femininity and Psychasthenia scores. A lower score on the Masculinity/Femininity scale of the hormonal therapy group may indicate that the transsexuals readily complied with cultural stereotypes of gender role. This finding is consistent with the literature^3^,^4^,^5^,^7^,^10^,^34^,^47^. We can hypothesize that participants with hormonal therapy have less need to assert themselves because of their bodies and their perception that their appearance is more in accordance with their stereotype. Previous authors have noted that the Masculinity/Femininity scale refers to stereotypical male and female interests and most likely reflects gender dysphoria^25^.

Hormonal therapy can be beneficial for anxiety and mood disorders^48^, which could explain why the individuals who were receiving hormonal therapy presented fewer disturbances on the Psychasthenia dimension compared with those who were not receiving hormonal therapy. It is now well established that, by inducing external physical modifications, regular hormone treatments produce a change in the gender role, greater self-confidence, greater comfort with others, and better social skills, and they reinforce gender affirmation through social recognition^44^,^49^,^50^. This finding is in line with recent studies suggesting the positive role of hormonal therapy in the quality of life of transsexuals^51^,^52^.

There were several strengths and limitations of this study:

1. This study compared psychological test data obtained from individuals approved for gender reassignment procedure with test data obtained from individuals who were rejected. This comparison showed higher scores among the non-eligible individuals for two clinical scales: Hysteria and Schizophrenia. The extrapolation of the results should consider these particularities. Selection bias could explain the relatively low levels of comorbidities. Future studies could focus on the individuals who do not meet criteria for gender reassignment procedure, do not want genital surgery, and who are outside of organized care systems.

2. The definitions of hormonal therapy and onset age should be discussed. In France, three months of hormonal therapy at low doses is often prescribed at the beginning of treatment, this is not enough to produce substantial physical/psychological changes. There is no generally single definition regarding the onset age of transsexual development. An early onset transsexual development could be defined as one in which the person fulfilled the DSM-V criteria for gender dysphoria in children^18^.

3. How the psychopathology profile is assessed warrants further discussion. The MMPI-2 is one of the best standardized methods for assessing the psychopathological profile of adult populations aged 18 years and over, including transsexual individuals^5^,^36^,^53^,^54^. The MMPI-2 was a major revision of the MMPI^23^ and was standardized on a new large national sample of adults in the United States. A wide variety of subscales was also introduced over many years to help clinicians interpret the results of the original clinical scales, which had been found to contain a general factor that made interpretation of scores on the clinical scales difficult^55^,^56^. The latest version of the MMPI-2, the MMPI-2-restructured form, could not be used because the beginning of this study preceded the availability of this version^57^. The use of this test on transsexual populations relies on: i) the availability of both female and male norms, ii) the identification of response distortion from the 3 validity scales, and iii) the availability of a specific Masculinity/Femininity clinical scale, which is especially pertinent to the assessment of transgender individuals.

4. Cultural influences and theoretical viewpoints may impact treatment and gender reassignment in France and future studies should consider this parameter to clearly understand/interpret the impact that the psychological profile of transsexuals has on therapeutic strategies. Indeed, while psychological distress can be inherent to being transsexual, it could also be socially induced by the society, peers and family^2^.

5. An assessment of depression, anxiety, and coping using specific and objective tools would be helpful to correlate our findings with these measures. Future studies should include these scales to support more valid clinical implications.

## Conclusion

A psychological profile assessment can help a multidisciplinary gender dysphoria team to detect potential psychopathological factors of vulnerability, changes and stabilization over time of the abilities of adaptability of the individual. Health professionals should screen for and incorporate co-existing psychopathological concerns into the treatment plan.

## Additional Information

**How to cite this article**: Bonierbale, M. *et al.* MMPI-2 Profile of French Transsexuals: The Role of Sociodemographic and Clinical Factors. A cross-sectional design. *Sci. Rep.* **6**, 24281; doi: 10.1038/srep24281 (2016).

## Figures and Tables

**Table 1:** Characteristics comparison between non-eligible (n = 29) and eligible (n = 114) individuals.

		Non-eligible n = 29 N (%)	Eligible n = 114 N (%)	P^*^
Sex	FtM	6 (20.7)	56 (49.1)	<*0.01*
	MtF	23 (79.3)	58 (50.9)	
Age	<31 years	11 (37.9)	54 (47.4)	*NS*
	≥31 years	18 (62.1)	60 (52.6)	
Hormons	Without	22 (75.9)	55 (49.1)	<*0.01*
	With	7 (24.1)	57 (50.9)	
MMPI-2	Invalid	2 (6.9)	6 (5.3)	*NS*
	Not invalid	27 (93.1)	108 (94.7)	
Clinical scales	≥65	%	%	p^*^
	Hs	22.2	17.6	*NS*
	D	11.1	14.8	*NS*
	Hy	33.3	13.0	<*0.05*
	Pd	33.3	23.1	*NS*
	Mf	51.9	56.5	*NS*
	Pa	25.9	17.6	*NS*
	Pt	18.5	16.7	*NS*
	Sc	22.2	13.9	*NS*
	Ma	7.4	11.1	*NS*
	Si	14.8	8.3	*NS*

*p-value.

NS non-significant.

Hs Hypochondriasis, D Depression, Hy Hysteria, Pd Psychopathic Deviate, Mf Masculinity/Femininity, Pa Paranoia, Pt Psychasthenia, Sc Schizophrenia, Ma Hypomania, and Si Social Introversion.

**Table 2:** Comparison of characteristics and MMPI scales according to the biological sex (n = 108).

		FtM (n = 54)	MtF (n = 54)	p			
Age (median)	<31 years	37 (68.5)	16 (29.6)	**<*****0.001***			
	≥31 years	17 (31.5)	38 (70.4)				
Hormonal therapy	Without	38 (71.7)	16 (30.2)	**<*****0.001***			
	With	15 (28.3)	37 (69.8)				
Onset age	Early	52 (100)	31 (57.4)	**<*****0.001***			
	Late	–	23 (42.6)				
Sexual orientation	Same biological sex	50 (98.0)	33 (64.7)	**<*****0.001***			
	Others	1 (2.0)	18 (35.3)				
		M ± SD	M ± SD	p	% ≥ 65	% ≥ 65	p
MMPI-2	Hs	49.0 ± 10.9	57.7 ± 11.0	**<*****0.001***	9.3	25.9	**<*****0.05***
	D	49.5 ± 11.6	56.0 ± 8.3	**<*****0.001***	7.4	22.2	**<*****0.05***
	Hy	50.0 ± 10.6	58.2 ± 10.0	**<*****0.001***	7.4	18.5	*NS*
	Pd	56.4 ± 11.0	57.7 ± 11.1	*NS*	20.4	25.9	*NS*
	Mf	65.2 ± 12.0	64.0 ± 9.1	*NS*	59.3	53.7	*NS*
	Pa	52.7 ± 10.2	58.1 ± 10.3	**<*****0.01***	13.0	22.2	*NS*
	Pt	51.4 ± 10.3	54.6 ± 11.8	*NS*	13.0	20.4	*NS*
	Sc	53,5 ± 9,6	55,4 ± 11,4	*NS*	13.0	14.8	*NS*
	Ma	52.8 ± 9.9	51.7 ± 11.0	*NS*	13.0	9.3	*NS*
	Si	52.1 ± 8.8	51.8 ± 9.4	*NS*	5.6	11.1	*NS*

M ± SD mean ± standard deviation, p p-value, bold values: p < 0.05, NS non significative.

FtM female-to-male, MtF male-to-female.

Hs Hypochondriasis, D Depression, Hy Hysteria, Pd Psychopathic Deviate, Mf Masculinity/Femininity, Pa Paranoia, Pt Psychasthenia, Sc Schizophrenia, Ma Hypomania, and Si Social Introversion.

**Table 3:** Comparison of the MMPI scores according to age group, hormonal therapy status, onset age, and sexual orientation in FtMs individuals (n = 54).

Age		Hs	D	Hy	Pd	Mf	Pa	Pt	Sc	Ma	Si
<31 years (37)	M ± SD	47.5 ± 9.9	49.3 ± 10.6	48.5 ± 8.8	55.5 ± 11.2	65.4 ± 11.3	52.3 ± 8.6	53.1 ± 10.1	53.4 ± 10.1	53.8 ± 11.0	52.9 ± 8.9
≥31 years (17)	M ± SD	52.1 ± 12.6	49.8 ± 13.8	53.2 ± 13.5	58.2 ± 10.8	64.8 ± 13.9	53.5 ± 13.2	47.9 ± 10.1	51.7 ± 8.3	50.7 ± 6.9	50.5 ± 8.8
p-value		*NS*	*NS*	*NS*	*NS*	*NS*	*NS*	*NS*	*NS*	*NS*	*NS*
<31 years (37)	% ≥ 65	5.4	8.1	2.7	18.9	64.9	13.5	13.5	16.2	16.2	5.4
≥31 years (17)	% ≥ 65	17.6	5.9	17.6	23.5	47.1	11.8	11.8	5.9	5.9	5.9
p-value		*NS*	*NS*	*NS*	*NS*	*NS*	*NS*	*NS*	*NS*	*NS*	*NS*
**Hormonal therapy**											
Without (38)	M ± SD	48.0 ± 10.0	49.8 ± 11.1	49.3 ± 9.9	57.7 ± 10.6	67.3 ± 12.0	53.7 ± 8.6	53.0 ± 10.4	55.5 ± 9.1	54.0 ± 11.2	53.2 ± 8.1
With (15)	M ± SD	51.3 ± 13.6	49.2 ± 13.5	52.0 ± 12.8	53.1 ± 12.3	59.3 ± 10.6	50.4 ± 13.7	46.9 ± 9.4	48.3 ± 9.3	49.6 ± 4.7	49.4 ± 10.6
p-value		*NS*	*NS*	*NS*	*NS*	<***0.05***	*NS*	*0.05*	<***0.01***	*NS*	*NS*
Without (38)	% ≥ 65	7.9	7.9	5.3	23.7	65.8	13.2	15.8	15.8	18.4	5.3
With (15)	% ≥ 65	13.3	6.7	13.3	13.3	40.0	13.3	6.7	6.7	0	6.7
p-value		*NS*	*NS*	*NS*	*NS*	*NS*	*NS*	*NS*	*NS*	*NS*	*NS*
**Onset age°**											
Early (52)	M ± SD	48.8 ± 10.8	49.3 ± 11.8	49.6 ± 10.1	55.9 ± 10.7	65.2 ± 12.2	52.4 ± 10.3	51.5 ± 10.5	53.3 ± 9.6	52.9 ± 10.1	51.9 ± 8.8
Early (52)	% ≥ 65	7.7	7.7	5.8	19.2	59.6	13.5	13.5	13.5	13.5	3.8
**Sexual orientation°**											
Same biological sex (50)	M ± SD	48.6 ± 10.9	48.5 ± 11.0	49.3 ± 10.1	55.5 ± 10.6	66.0 ± 11.7	52.3 ± 10.4	50.9 ± 9.8	52.9 ± 9.1	53.0 ± 10.3	51.4 ± 8.2
Others (1)		55	81	64	76	40	62	79	79	52	77
Same biological sex (50)	% ≥ 65	8.0	6	6.0	18.0	62.0	14.0	12.0	12.0	14.0	2.0
Others (1)	% ≥ 65	0	100	0	100	0	0	100	100	0	100

M ± SD mean ± standard deviation, p p-value, bold values: p < 0.05.

Hormonal therapy: ‘With’: individuals had received cross-sex hormones for a minimum of 3 months’ duration prescribed by a physician as a part of the sex reassignment procedure; ‘Without’: all other cases, corresponding to a minimal delay for expected physical changes.

Onset age: ‘Early’: individuals with onset age beginning before puberty, ‘Late’: individuals with onset age beginning after puberty.

Hs Hypochondriasis, D Depression, Hy Hysteria, Pd Psychopathic Deviate, Mf Masculinity/Femininity, Pa Paranoia, Pt Psychasthenia, Sc Schizophrenia, Ma Hypomania, and Si Social Introversion.

°No statistical performed.

**Table 4:** Comparison of the MMPI scores according to age group, hormonal therapy status, onset age, and sexual orientation in MtFs individuals (n = 54).

Age		Hs	D	Hy	Pd	Mf	Pa	Pt	Sc	Ma	Si
<31 years (16)	M ± SD	52.5 ± 8.3	53.6 ± 8.3	54.5 ± 10.7	55.9 ± 13.4	62.9 ± 9.8	60.4 ± 12.2	54.3 ± 13.6	55.0 ± 14.5	56.4 ± 14.9	51.4 ± 10.2
≥31 years (38)	M ± SD	59.9 ± 11.3	57.0 ± 8.3	59.8 ± 9.4	58.4 ± 10.1	64.5 ± 8.9	57.1 ± 9.3	54.7 ± 11.1	55.5 ± 10.1	49.7 ± 8.4	52.0 ± 9.1
p-value		**<*****0.05***	*NS*	*NS*	*NS*	*NS*	*NS*	*NS*	*NS*	*NS*	*NS*
<31 years (16)	% ≥ 65	18.8	12.5	12.5	31.3	50.0	37.5	25.0	18.8	18.8	18.8
≥31 years (38)	% ≥ 65	28.9	26.3	21.1	23.7	55.3	15.8	18.4	13.2	5.3	7.9
p-value		*NS*	*NS*	*NS*	*NS*	*NS*	*NS*	*NS*	*NS*	*NS*	*NS*
**Hormonal therapy**											
Without (16)	M ± SD	60.0 ± 11.7	56.7 ± 8.7	60.1 ± 11.4	59.5 ± 14.3	64.5 ± 7.1	59.9 ± 12.0	58.9 ± 15.7	59.1 ± 14.8	57.6 ± 13.3	55.2 ± 9.4
With (37)	M ± SD	56.1 ± 10.7	55.7 ± 8.4	56.9 ± 8.8	56.5 ± 9.3	64.0 ± 9.9	57.2 ± 9.6	52.8 ± 9.4	53.7 ± 9.6	49.0 ± 8.7	50.4 ± 9.2
p-value		*NS*	*NS*	*NS*	*NS*	*NS*	*NS*	*NS*	*NS*	**<*****0.05***	*NS*
Without (16)	% ≥ 65	37.5	25.0	31.3	37.5	62.5	25.0	43.8	25.0	18.8	18.8
With (37)	% ≥ 65	18.9	21.6	10.8	18.9	51.4	21.6	10.8	10.8	5.4	8.1
p-value		*NS*	*NS*	*NS*	*NS*	*NS*	*NS*	**<*****0.05***	*NS*	*NS*	*NS*
**Onset age**											
Early (31)	M ± SD	56.3 ± 10.3	55.0 ± 8.7	55.7 ± 9.7	56.3 ± 11.6	63.3 ± 10.3	59.1 ± 11.4	55.4 ± 12.4	55.6 ± 13.3	53.8 ± 11.0	53.0 ± 11.2
Late (23)	M ± SD	59.7 ± 11.7	57.2 ± 7.8	61.7 ± 9.6	59.6 ± 10.3	65.0 ± 7.2	56.7 ± 8.6	53.5 ± 11.0	55.0 ± 8.5	48.8 ± 10.5	50.2 ± 6.1
p-value		*NS*	*NS*	*NS*	*NS*	*NS*	*NS*	*NS*	*NS*	**<*****0.05***	*NS*
Early (31)	% ≥ 65	22.6	19.4	16.1	25.8	48.4	29.0	22.6	19.4	9.7	19.4
Late (23)	% ≥ 65	30.4	26.1	21.7	26.1	60.9	13.0	17.4	8.7	8.7	0
p-value		*NS*	*NS*	*NS*	*NS*	*NS*	*NS*	*NS*	*NS*	*NS*	**<*****0.05***
**Sexual orientation**											
Same biological sex (33)	M ± SD	57.8 ± 11.6	56.1 ± 8.7	57.4 ± 10.6	57.5 ± 11.6	64.2 ± 9.8	60.1 ± 10.7	56.9 ± 12.5	56.3 ± 13.9	54.3 ± 11.6	53.1 ± 10.0
Others (18)	M ± SD	56.8 ± 10.6	55.1 ± 7.8	58.4 ± 8.6	58.6 ± 9.9	63.9 ± 7.8	54.7 ± 8.1	51.4 ± 10.3	54.1 ± 5.7	47.6 ± 8.9	49.3 ± 8.4
p-value		*NS*	*NS*	*NS*	*NS*	*NS*	*NS*	*NS*	*NS*	**<*****0.05***	*NS*
Same biological sex (33)	% ≥ 65	27.3	24.2	21.2	30.3	57.6	30.3	27.3	24.2	12.1	18.2
Others (18)	% ≥ 65	16.7	16.7	11.1	16.7	50.0	5.6	11.1	0	5.6	0
p-value		*NS*	*NS*	*NS*	*NS*	*NS*	*NS*	*NS*	**<*****0.05***	*NS*	*NS*

M ± SD mean ± standard deviation, p p-value, bold values: p < 0.05.

Hormonal therapy: ‘With’: individuals had received cross-sex hormones for a minimum of 3 months’ duration prescribed by a physician as a part of the sex reassignment procedure; ‘Without’: all other cases, corresponding to a minimal delay for expected physical changes.

Onset age: ‘Early’: individuals with onset age beginning before puberty, ‘Late’: individuals with onset age beginning after puberty.

Hs Hypochondriasis, D Depression, Hy Hysteria, Pd Psychopathic Deviate, Mf Masculinity/Femininity, Pa Paranoia, Pt Psychasthenia, Sc Schizophrenia, Ma Hypomania, and Si Social Introversion.
